# Beyond Resection: Determinants of Outcome After Pediatric Brain Arteriovenous Malformation Surgery

**DOI:** 10.3390/medicina62091679

**Published:** 2026-09-01

**Authors:** Goran Tasić, Aleksandar Janićijević, Jelena Kostić, Aleksandar Milosavljević, Nikola Jovićević, Nikola Repac, Igor Nikolić, Ana Tasić, Maša Radovanović, Vladimir Jovanović

**Affiliations:** 1 Department for Cerebrovascular Diseases and Vascular Anomalies, Clinic of Neurosurgery, University Clinical Centre of Serbia, 11000 Belgrade, Serbia; angie3bgbg@yahoo.com (G.T.); aleksandarjanicijevic82@gmail.com (A.J.); n.jovicevic05@gmail.com (N.J.); nyjaphen@yahoo.com (N.R.); i.m.nikolic@gmail.com (I.N.); dr.aleksandar.janicijevic@gmail.com (V.J.); 2 Faculty of Medicine, University of Belgrade, 11000 Belgrade, Serbia; 3 Clinic of Neurosurgery, University Clinical Center of Kragujevac, 34000 Kragujevac, Serbia; milosavljevic0410@gmail.com; 4 Faculty of Medical Science, University of Kragujevac, 34000 Kragujevac, Serbia; 5 University Children’s Hospital, University Clinical Center of Serbia, 11000 Belgrade, Serbia; ana.gtasic@gmail.com (A.T.); r.masilija@gmail.com (M.R.)

**Keywords:** arteriovenous malformation, pediatric neurosurgery, microsurgical resection, intraventricular hemorrhage, modified Rankin Scale, Spetzler–Martin grade, functional outcome, cerebrovascular disease

## Abstract

*Background and Objectives*: brain arteriovenous malformations (AVMs) are a leading cause of spontaneous intracranial hemorrhage in children and carry a disproportionately high lifetime hemorrhagic risk. *Materials and Methods*: We retrospectively analyzed clinical, morphological, and surgical factors associated with functional outcome in 30 consecutive pediatric patients (≤18 years) who underwent microsurgical AVM resection at a single tertiary neurosurgical center between January 2015 and December 2025. *Results*: Functional status was assessed with the modified Rankin Scale (mRS) at hospital discharge and at 6–9 months of follow-up; the Wilcoxon signed-rank test, Mann–Whitney U test, Fisher’s exact test, Spearman correlation, and univariable logistic regression were used for analysis. Complete resection on early postoperative CT angiography was documented in all 30 patients (100%). The proportion with a favorable outcome (mRS 0–2) improved significantly from 50.0% at discharge to 76.7% at follow-up (*p* < 0.0001); 80.8% of survivors improved by at least one mRS point, and none deteriorated. In univariable analysis, the variable most strongly associated with poor outcome was intraventricular hemorrhage (IVH; odds ratio [OR] 28.5, 95% CI 2.65–306.6; *p* = 0.002); the wide confidence interval reflects the small number of events and precludes a precise estimate of effect size. Lower admission Glasgow Coma Scale (GCS) score (*p* = 0.0006), higher Supplemented Spetzler–Martin (S–M) grade (*p* = 0.019), and postoperative complications (*p* = 0.016) were likewise associated with poor outcome. Because these associations are univariable and unadjusted for confounding, they should be regarded as exploratory and hypothesis-generating rather than as independent predictors. All four deaths occurred in malformations in eloquent locations (*p* = 0.037). Microsurgical resection achieved complete resection on early postoperative CT angiography in all patients; however, functional recovery appeared to be associated primarily with the severity of the initial hemorrhagic insult—particularly IVH—and with lesion complexity rather than with the achievement of angiographic resection alone. *Conclusions*: Given the small, surgically selected cohort and the limited follow-up, these findings require confirmation in larger multicenter studies.

## 1. Introduction

Brain arteriovenous malformations (AVMs) are congenital vascular lesions characterized by arterial blood flow from afferent arteries through the nidus of dysplastic blood vessels into the venous drainage system. In the pediatric population, brain AVMs are among the leading causes of spontaneous intracranial hemorrhage and are associated with substantial long-term neurological morbidity. Hemorrhagic events constitute the most frequent mode of presentation, although seizures and progressive focal deficits may also occur [1,2]. Unlike adults, pediatric patients remain exposed to the risk of hemorrhage over a much longer period of time, resulting in a substantially higher cumulative lifetime risk [3]. The annual hemorrhage risk in unruptured AVMs is approximately 2–4%, but the lifetime cumulative risk is substantially amplified in pediatric patients given their prolonged life expectancy [3].

Furthermore, recent evidence indicates that rupture often occurs early in life, highlighting the aggressive natural history of pediatric AVMs [4].

Management of pediatric AVMs remains challenging and requires an individualized approach based on lesion characteristics and clinical presentation. Current treatment options include microsurgical resection, endovascular embolization, stereotactic radiosurgery, or combined strategies [5]. Among these, microsurgical resection remains the only modality capable of providing immediate and complete eradication of the nidus. When total removal is achieved, the risk of future hemorrhage is substantially reduced and an immediate, durable cure can be anticipated in most patients [6,7,8]; in children, however, this protection cannot be regarded as absolute, since delayed recurrence has been documented even after angiographically confirmed complete resection, as outlined below. In contrast, non-surgical approaches are limited by delayed therapeutic effect and persistent exposure to hemorrhagic risk.

Pediatric patients exhibit a pronounced capacity for neurological recovery following treatment, which is commonly attributed to the plasticity of the developing brain. This is supported by studies demonstrating more favorable outcomes in children compared with adults after surgical resection [9]. However, surgery is not without risk, particularly in cases involving eloquent or deep-seated brain regions, where the likelihood of postoperative deficits is increased [10]. Therefore, treatment decisions must carefully balance the potential benefits of complete lesion eradication against the risk of procedure-related morbidity.

Recent advances in imaging techniques, intraoperative navigation, and microsurgical strategies have improved the safety and effectiveness of AVM surgery [5,6,7]. At the same time, increasing awareness of recurrence in pediatric patients underscores the need for long-term follow-up [11,12]. Despite these advances, optimal management strategies continue to evolve, and further outcome data remain essential.

Recurrence following angiographically confirmed complete resection represents an additional pediatric-specific concern, with reported rates of 2–10% even after complete resection, likely reflecting ongoing vascular remodeling in the immature cerebrovascular system [13].

The objective of the present study was to retrospectively analyze the clinical, morphological, and surgical factors associated with functional outcome in a consecutive series of pediatric patients undergoing microsurgical resection for cerebral AVM at a single tertiary neurosurgical center, with a specific focus not only on complete resection but also on the factors associated with long-term neurological outcome. Also, to our knowledge, no dedicated pediatric AVM microsurgical series has previously been reported from the Western Balkans region, making this the first such institutional experience from this geographic area.

## 2. Materials and Methods

### 2.1. Study Design and Patient Selection

This was a retrospective single-center study of 30 pediatric patients (<18 years of age at presentation) with ruptured or unruptured cerebral AVM who underwent microsurgical resection alone at the Department for Cerebrovascular Diseases and Vascular Anomalies, Clinic of Neurosurgery, University Clinical Centre of Serbia, between January 2015 and December 2025. Inclusion criteria were age < 18 years at presentation, confirmed cerebral AVM on neuroimaging, microsurgical resection as the primary treatment modality, complete clinical data, and total resection confirmed by computed tomography angiography (CTA). Patients receiving primary radiosurgery or endovascular embolization as definitive treatment were excluded, as were those with multimodality treatment. Consequently, the cohort reflects a surgically selected population rather than the full spectrum of pediatric AVMs, and the results should be interpreted specifically within this context rather than generalized to children managed with radiosurgery, embolization, or multimodality strategies.

### 2.2. Neuroimaging and Lesion Characterization

All patients underwent pre- and postoperative neuroimaging, including computed tomography (CT), CTA, and/or digital subtraction angiography (DSA). Nidus size was categorized as small (<3 cm), medium (3–6 cm), or large (>6 cm). Lesion complexity was assessed using the Supplemented Spetzler–Martin (S–M) grading system [14]. Venous drainage was classified as superficial, deep, or combined. Postoperative resection was confirmed by CTA on postoperative day 1–5. CTA was adopted as the routine early postoperative confirmation modality because it is less invasive, more readily available in the early postoperative setting, and avoids the arterial access and radiation burden of catheter angiography in children; digital subtraction angiography (DSA), which remains the reference standard for confirming AVM obliteration, was reserved for cases with equivocal CTA findings and was not performed systematically in every patient. This strategy may have reduced sensitivity for small residual nidus components, a limitation addressed further in Section 4.7.

### 2.3. Outcome Measures

Functional outcome was assessed using the modified Rankin Scale (mRS) at hospital discharge and at 6–9 months of postoperative follow-up. Outcomes were dichotomized as favorable (mRS 0–2, functional independence) and unfavorable (mRS 3–6, dependency or death). Because of the retrospective design, mRS scores were assigned by the treating neurosurgeons from the clinical documentation rather than prospectively by an independent blinded assessor; formal interobserver agreement was therefore not evaluated, which represents a potential source of assessment bias. The 6–9-month interval reflects the duration of structured functional follow-up and is comparatively short for pediatric AVMs, in which angiographic recurrence may become apparent only years after apparently complete resection. Postoperative complications were classified as postoperative intracranial hemorrhage, infectious complications (meningitis and/or systemic infection), permanent new neurological deficit, and death.

### 2.4. Statistical Analysis

Statistical analysis was performed using IBM SPSS Statistics version 26.0 (IBM Corp., Armonk, NY, USA). Continuous variables are reported as mean ± standard deviation (SD) or median with interquartile range (IQR). Categorical variables are expressed as absolute frequencies and percentages. The Wilcoxon signed-rank test was applied to compare paired mRS scores between discharge and 6–9 months of follow-up. The Spearman rank correlation coefficient (ρ) was used to assess monotonic associations between continuous variables. The Mann–Whitney U test compared mRS distributions between independent groups. Fisher’s exact test was applied for categorical associations. Univariable binary logistic regression was used to estimate odds ratios (OR) with 95% confidence intervals (CI) for the association between individual baseline variables and a poor functional outcome (mRS 3–6); for categorical predictors the reported *p*-values were derived from Fisher’s exact test, whereas for continuous predictors group differences were assessed with the Mann–Whitney U test. Because several predictors had a low number of events—with quasi-complete data separation for intraventricular hemorrhage—the affected odds ratios have very wide confidence intervals and are reported as unstable, exploratory estimates rather than precise measures of effect. When a variable was not documented for every patient (for example, admission GCS was available in 28 of 30 patients), the available-case denominator was used and is stated explicitly. All tests were two-tailed; *p* < 0.05 was considered statistically significant. Associations with *p* ≥ 0.05 are reported as non-significant trends and are not interpreted as evidence of an effect. Due to the limited sample size (*n* = 30), multivariable regression analysis was not feasible; all associations reflect univariable relationships. No formal a priori sample-size or power calculation was performed; given the small cohort (*n* = 30) and the low number of outcome events, the study is likely underpowered to detect clinically meaningful associations, particularly in the mortality and secondary analyses. Because multiple univariable comparisons were performed, the risk of false-positive findings is increased; no formal correction for multiple testing was applied, and all reported associations should therefore be regarded as exploratory and hypothesis-generating.

## 3. Results

During the study period, 30 consecutive pediatric patients (aged ≤18 years) with an angiographically confirmed cerebral AVM were treated with microsurgical resection and included in the analysis. Univariable logistic regression was used to estimate odds ratios (OR) with 95% confidence intervals (CI). Where individual fields were not documented in the source registry, the analytic denominator is stated explicitly.

### 3.1. Baseline Demographics and Clinical Presentation

**Figure 1:**
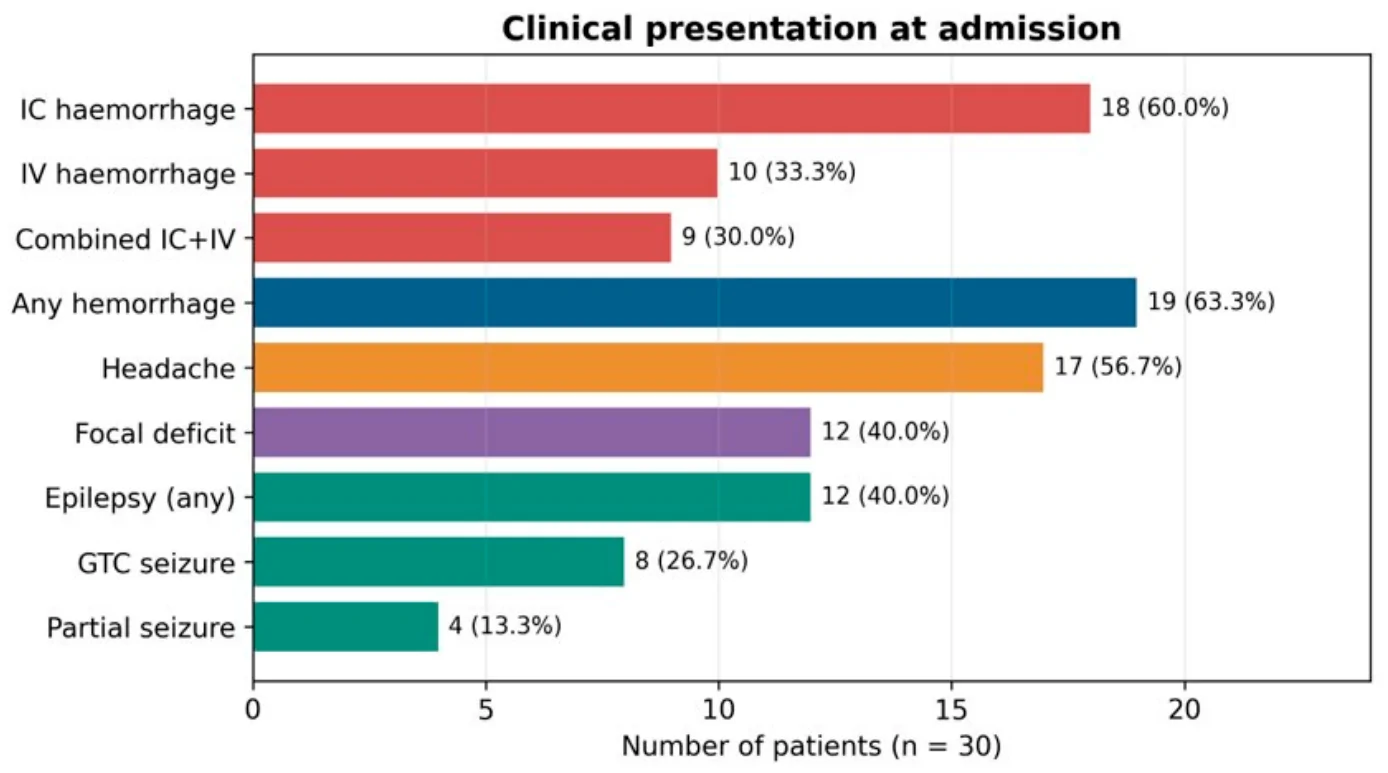
Frequency of the principal clinical presentations at admission. A hemorrhagic mode of onset dominated the cohort, affecting nearly two-thirds of patients, whereas seizures and headache accounted for the majority of non-hemorrhagic presentations. Percentages are calculated over the full cohort (*n* = 30); categories are non-mutually exclusive.

The cohort comprised 18 boys (60.0%) and 12 girls (40.0%), with a mean age of 13.6 ± 3.7 years (median 14; IQR 11.3–17.0; range 4–18). A hemorrhagic mode of presentation predominated and was documented in 19 patients (63.3%). Intracerebral hemorrhage (ICH) occurred in 18 patients (60.0%), intraventricular hemorrhage (IVH) in 10 (33.3%), and a combined intracerebral–intraventricular pattern in 9 (30.0%). Epileptic seizures were the presenting feature in 12 patients (40.0%)—generalized tonic–clonic in 8 (26.7%) and partial in 4 (13.3%)—while headache was reported by 17 patients (56.7%) and a focal neurological deficit by 12 (40.0%). The GCS on admission was 15 in the majority of patients (median 15; IQR 13.75–15.0); however, 6 of the 28 patients with a documented score (21.4%) presented in the severe range (GCS 3–8), reflecting the clinical impact of the hemorrhagic ictus. Baseline characteristics are summarized in Table 1, and the frequency of the principal clinical presentations at admission is shown in Figure 1.

### 3.2. Angiomorphological and Angioarchitectural Characteristics

Lateralization was balanced, with 16 right-sided (53.3%) and 14 left-sided (46.7%) malformations. By maximal nidus diameter, 14 AVMs (46.7%) were small (<3 cm), 13 (43.3%) were medium (3–6 cm), and 2 (6.7%) were large (>6 cm); the nidus size category was not documented in one patient. The Supplemented S–M grade ranged from 2 to 8 (median 3.5; mean 3.9), with grade 3 being the single most frequent value (13 patients, 43.3%). An eloquent nidus location—motor cortex, deep, cerebellar, or brainstem—was present in 14 patients (46.7%). By lobe, the frontal lobe was most frequently involved (eight patients; 26.7%), followed by the parietal (seven patients; 23.3%), occipital (six patients; 20.0%), and a deep compartment (six patients; 20.0%); a motor-cortex nidus was identified in four patients (13.3%), temporal in three (10.0%), cerebellar in three (10.0%), purely ventricular in two (6.7%), and brainstem in one (3.3%).

**Figure 2:**
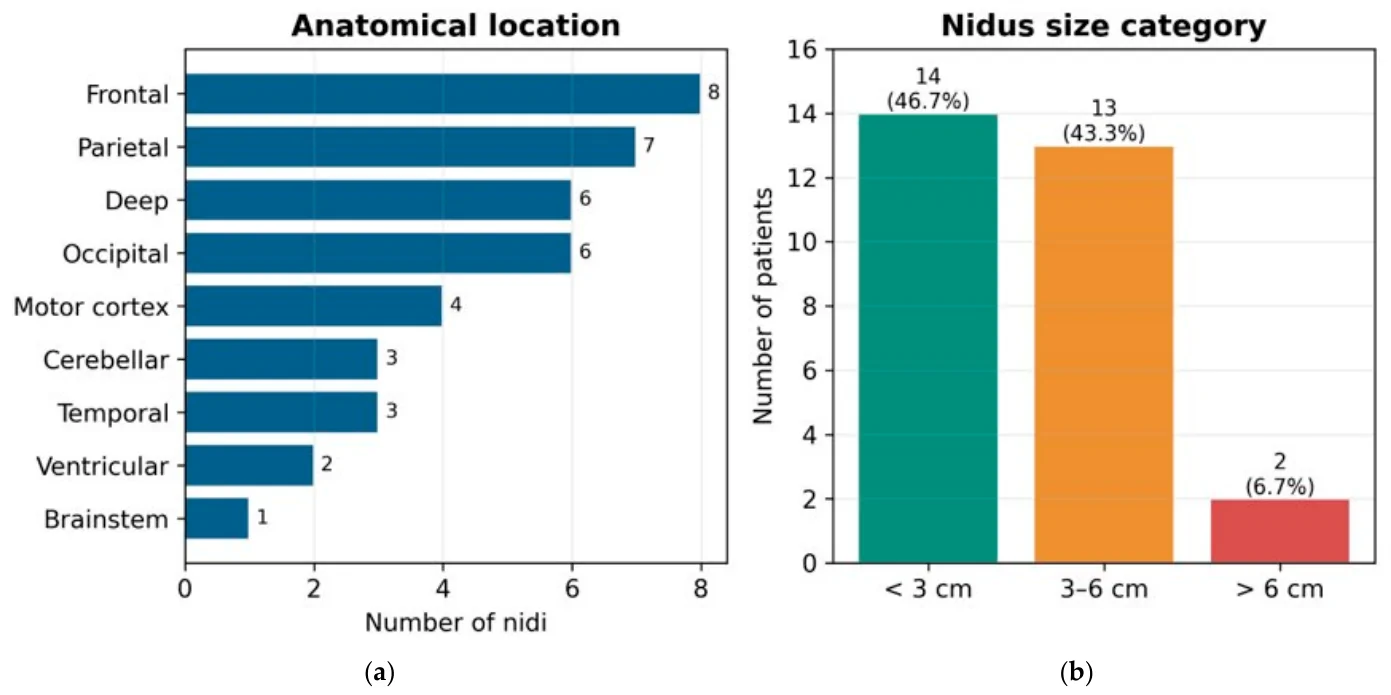
Anatomical distribution and size profile of the nidus. (**a**) Lobar and deep-compartment involvement, showing a supratentorial and predominantly frontal preponderance. (**b**) Nidus size category; 90% of malformations were ≤6 cm in diameter.

**Figure 3:**
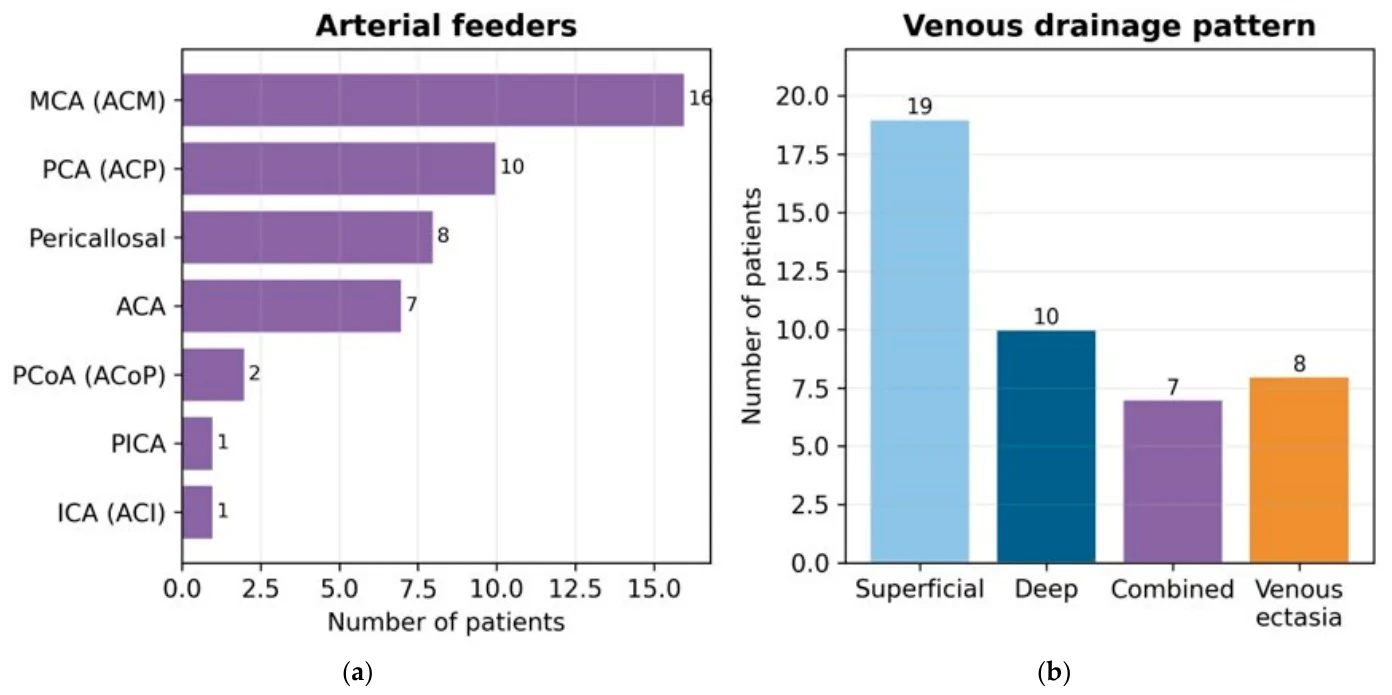
Angioarchitecture of the cohort. (**a**) Arterial feeder territories, dominated by the MCA and PCA. (**b**) Venous drainage pattern; a deep or combined venous component was present in 43.3% of patients.

Angioarchitecturally, the number of arterial feeders was documented in 24 patients: a single feeder in 10, two feeders in 10, and three feeders in 4 (mean 1.75). The middle cerebral artery (MCA) was the dominant supplier (16; 66.7%), followed by the posterior cerebral artery (PCA; 10; 41.7%), the pericallosal artery (8; 33.3%), and the anterior cerebral artery (ACA; 7; 29.2%). Venous drainage was assessable in 27 patients; a superficial component was present in 19 of 27, a deep component in 10 of 27, and a combined superficial–deep pattern in 7 of 26. These categories are not mutually exclusive, since a single malformation may drain by more than one route; a deep or combined venous component—the configuration carried forward into the outcome analysis—was present in 13 patients (13/30, 43.3%). Venous ectasia was identified in 8 of the 21 patients in whom it was explicitly assessed. Full angioarchitectural data are presented in Table 2; the anatomical distribution and size profile of the nidus and the angioarchitecture of the cohort are illustrated in Figure 2 and Figure 3, respectively.

### 3.3. Surgical Management and Postoperative Complications

All 30 patients (100%) underwent microsurgical resection of the nidus as the definitive treatment. Concomitant evacuation of an intracerebral hematoma was required in 13 patients (43.3%), an external ventricular drain was placed in 4 (13.3%), and a ventriculoperitoneal shunt was ultimately required in 3 (10.0%). At least one postoperative complication occurred in six patients (20.0%). The most frequent complication was postoperative intracerebral re-hemorrhage in five patients (16.7%), followed by meningitis and surgical-site infection in two each (6.7%), and shunt malfunction in one (3.3%). Surgical data are detailed in Table 3.

### 3.4. Functional Outcomes

**Figure 4:**
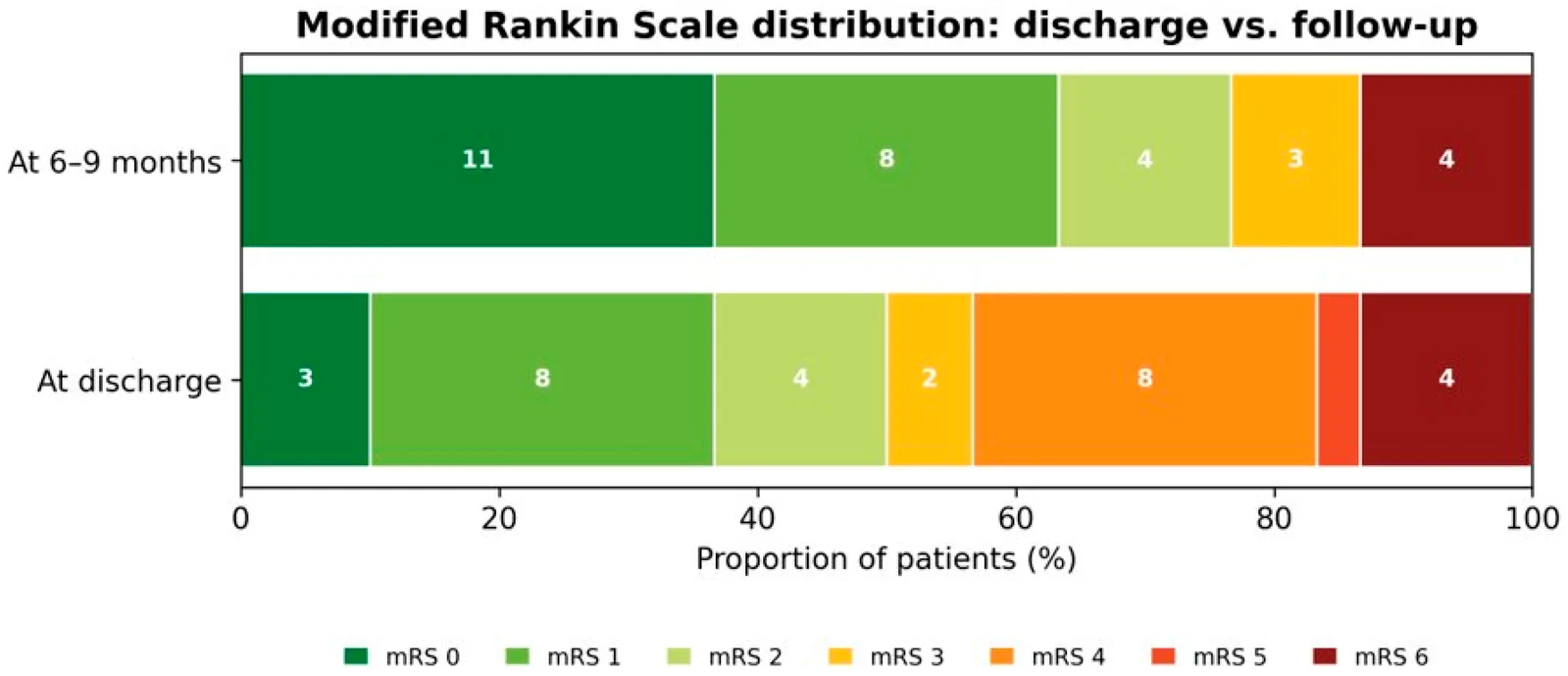
Modified Rankin Scale (mRS) shift plot comparing functional distribution at discharge and at 6–9 months. The favorable band (mRS 0–2) expanded from 50.0% to 76.7%; the moderate-disability tier (mRS 4–5) had essentially resolved by follow-up.

**Figure 5:**
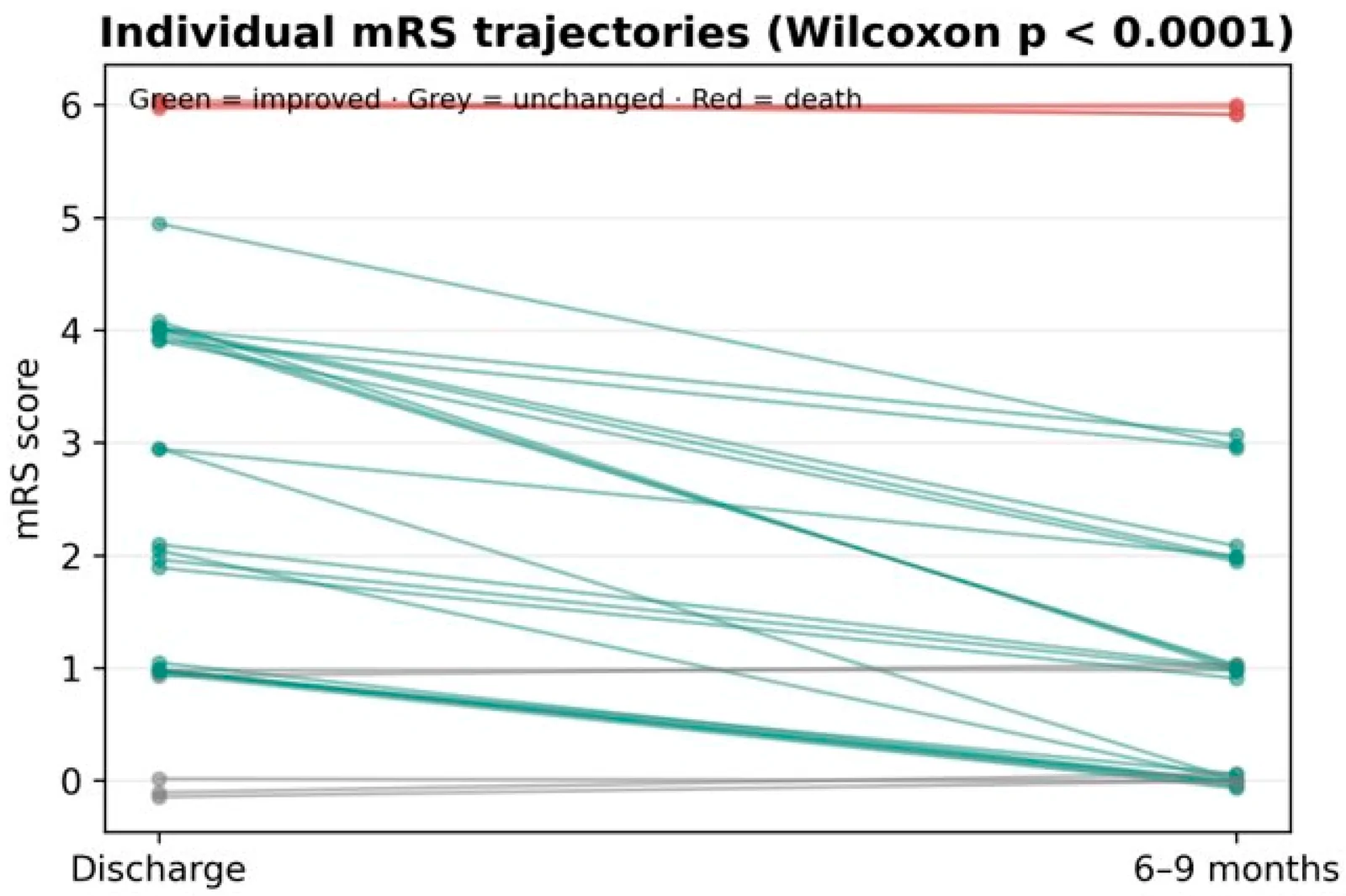
Individual patient mRS trajectories from discharge to 6–9 months. Green lines indicate functional improvement, grey lines unchanged status, and red lines the four deaths. No surviving patient deteriorated (Wilcoxon signed-rank *p* < 0.0001).

Functional status was graded with the mRS at discharge and at 6–9 months of follow-up; the four perioperative deaths (mRS 6) were carried forward as mRS 6 at follow-up. At discharge, 15 patients (50.0%) had a favorable outcome (mRS 0–2), 11 (36.7%) a poor outcome (mRS 3–5), and 4 (13.3%) had died. By 6–9 months, the proportion with a favorable outcome had risen markedly to 23 patients (76.7%), with 11 scoring mRS 0, 8 scoring mRS 1, and 4 scoring mRS 2; a poor outcome (mRS 3–6) persisted in 7 patients (23.3%), giving an overall mortality of 13.3%. The mRS distribution is presented in Table 4 and Figure 4, and individual patient mRS trajectories are shown in Figure 5.

The Wilcoxon signed-rank test demonstrated a highly significant reduction in mRS from discharge to follow-up (*p* < 0.0001). Among the 26 survivors, 21 (80.8%) improved by at least one mRS point, 5 (19.2%) were unchanged, and none deteriorated, underscoring the remarkable recovery potential of the pediatric brain after complete microsurgical resection.

### 3.5. Variables Associated with Poor Functional Outcome

**Figure 6:**
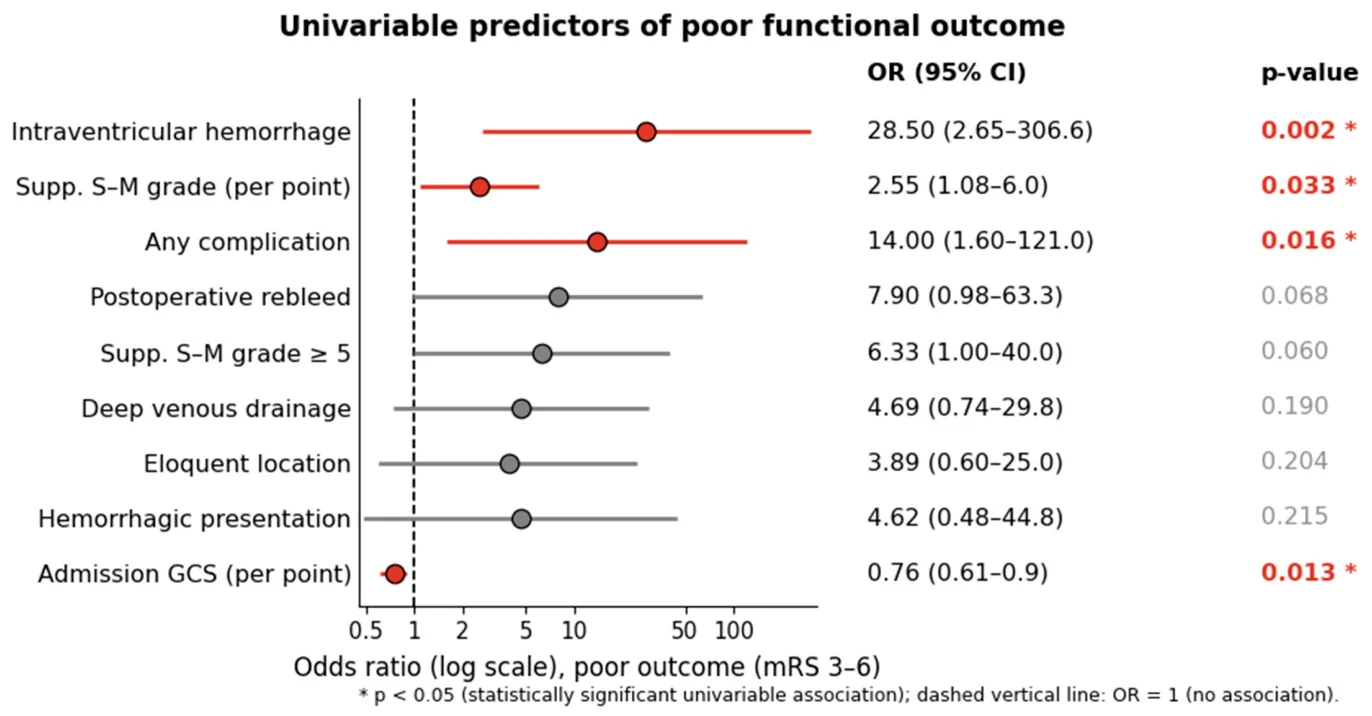
Forest plot of univariable odds ratios (log scale) for poor functional outcome (mRS 3–6). Variables reaching statistical significance in univariable testing (*p* < 0.05) are highlighted in red; these represent unadjusted associations rather than independent predictors. Intraventricular hemorrhage showed the strongest association with poor outcome, whereas each additional admission GCS point was associated with lower odds of poor outcome (OR 0.76); odds ratios for the continuous variables (admission GCS and Supplemented Spetzler–Martin grade) are expressed per one-unit increase, whereas those for categorical variables are per presence of the factor. Horizontal bars denote 95% confidence intervals; the dashed line marks OR = 1. The extremely wide confidence intervals for several estimates, most notably IVH, visually underscore the exploratory nature of these findings and the imprecision arising from the small number of events.

**Figure 7:**
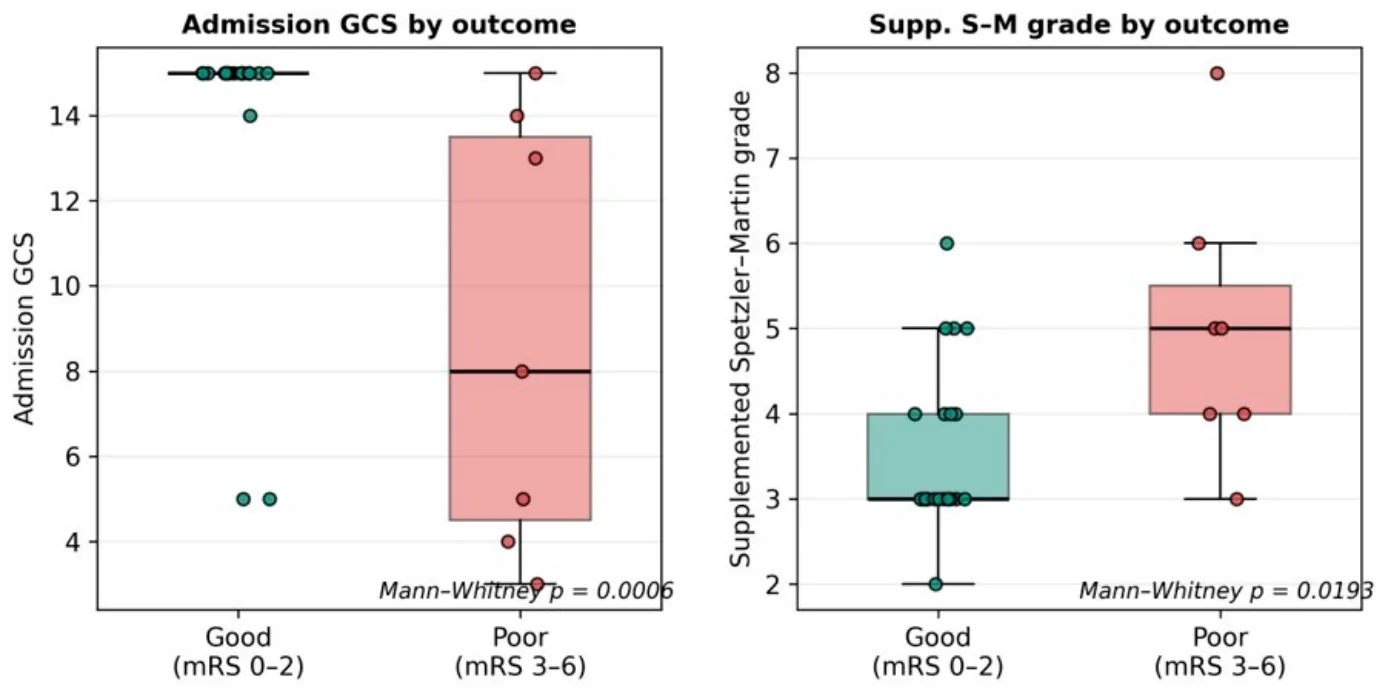
Distribution of admission GCS (**left panel**) and Supplemented Spetzler–Martin grade (**right panel**) stratified by 6–9-month outcome. Boxes show the median and interquartile range with individual data points overlaid; both metrics were significantly worse in the poor-outcome group.

Univariable analysis identified several variables associated with a poor functional outcome (mRS 3–6) at 6–9 months (Table 5; Figure 6); the distribution of admission GCS and Supplemented S–M grade stratified by outcome is shown in Figure 7. The categorical variable most strongly associated with poor outcome was IVH, which was present in 6 of the 7 poor-outcome patients versus 4 of the 23 favorable-outcome patients (OR 28.5; Fisher’s exact *p* = 0.002). A lower admission GCS was likewise powerfully associated with a poor outcome (median 8 vs. 15; Mann–Whitney *p* = 0.0006), and a higher Supplemented S–M grade discriminated the two groups (median 5 vs. 3; *p* = 0.0193). The occurrence of any postoperative complication was also significantly associated with poor outcome (OR 14.0; *p* = 0.0157), whereas postoperative re-hemorrhage showed a non-significant trend in the same direction (OR 7.9; 95% CI 0.98–63.3; Fisher’s exact *p* = 0.068). A Supplemented S–M grade ≥ 5 showed a strong trend (OR 6.33; *p* = 0.0596), whereas deep venous drainage, eloquent location, and hemorrhagic presentation, although associated with point estimates in the adverse direction (OR 3.9–4.7), did not reach significance in this small sample.

Univariable logistic regression corroborated these findings: each one-point decrement in admission GCS increased the odds of a poor outcome (OR 0.76 per point; 95% CI 0.61–0.94; *p* = 0.013), each one-grade increment in the Supplemented S–M scale raised them (OR 2.55; 95% CI 1.08–6.03; *p* = 0.033), while for IVH the quasi-complete separation of the data (present in 6 of 7 poor-outcome versus 4 of 23 favorable-outcome patients) precluded a stable maximum-likelihood estimate, so the association is reported from the corresponding 2 × 2 table (OR 28.5; 95% CI 2.65–306.6; Fisher’s exact *p* = 0.002). Spearman correlations were concordant: GCS was inversely correlated with the 6–9-month mRS (ρ = −0.65; *p* = 0.0002) and the Supplemented S–M grade was positively correlated (ρ = +0.52; *p* = 0.0045). These odds ratios should be interpreted with caution: for IVH in particular, the very wide confidence interval (95% CI 2.65–306.6) reflects the small number of events rather than a precise estimate of effect size, and the same caveat applies to the other categorical estimates.

### 3.6. Variables Associated with Mortality and Early (Discharge) Status

**Figure 8:**
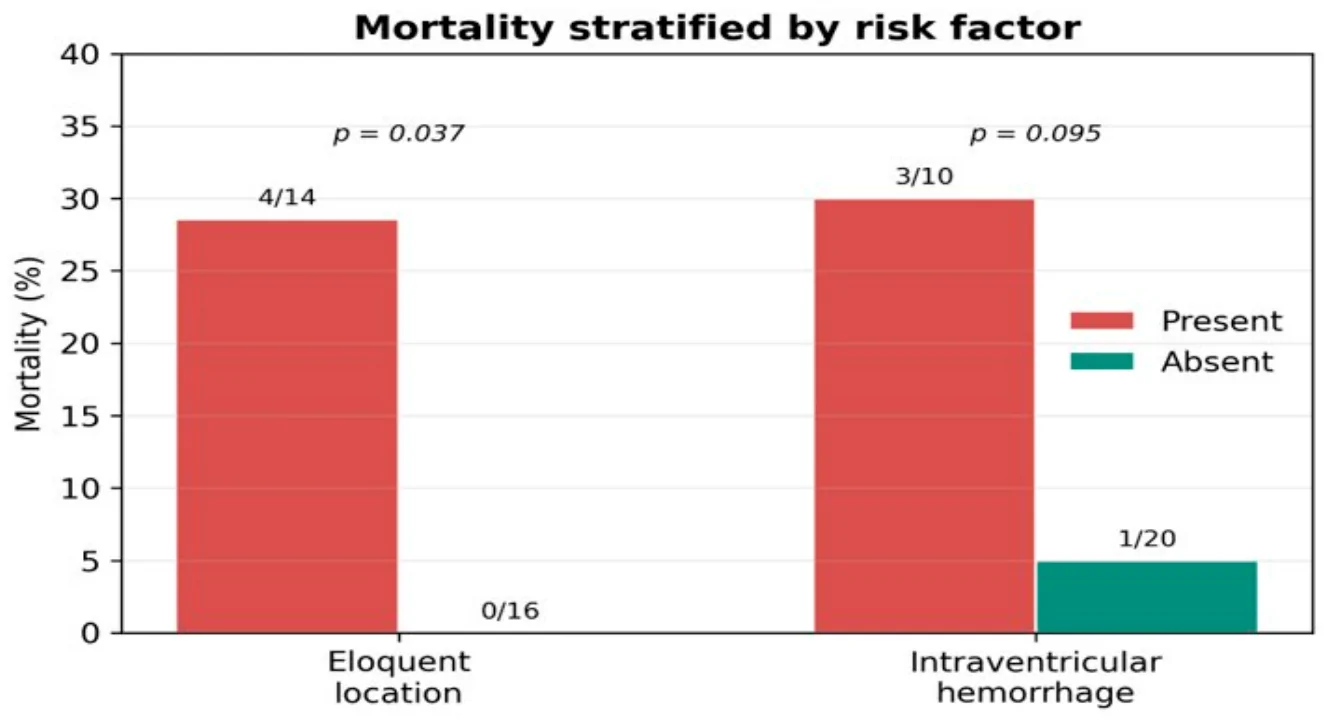
Perioperative mortality stratified by the two leading risk factors. Mortality was confined to eloquent-territory malformations (4/14 vs. 0/16; *p* = 0.037) and was numerically higher in the presence of IVH (3/10 vs. 1/20; *p* = 0.095). Labels above bars show deaths/total in each subgroup.

Four patients (13.3%) died in the perioperative period (Table 6; Figure 8). Mortality clustered entirely within eloquent-territory malformations: all 4 deaths occurred among the 14 patients with an eloquent nidus, versus none of the 16 patients with a non-eloquent nidus (*p* = 0.0365). A lower admission GCS was again significant (median 9 vs. 15; Mann–Whitney *p* = 0.0324), while IVH (OR 8.14; *p* = 0.0952) and a higher Supplemented S–M grade (*p* = 0.101) showed adverse trends without reaching significance. Because these analyses are based on only four deaths, all mortality estimates are statistically unstable, and the association between eloquent location and mortality in particular should be regarded as an exploratory observation rather than as evidence of an independent prognostic factor.

Analysis of early (discharge) functional status reinforced the role of the hemorrhagic ictus. A hemorrhagic presentation was strongly associated with poor status at discharge (13/19 vs. 2/11; OR 9.75; *p* = 0.0209), as was lower admission GCS (mean 10.8 vs. 14.9; *p* = 0.0083); IVH (OR 7.43; *p* = 0.0502) and a focal admission deficit (OR 6.0; *p* = 0.0604) did not reach statistical significance. The loss of significance of hemorrhagic presentation at 6–9 months is consistent with the strong functional recovery observed during follow-up.

### 3.7. Summary of Key Findings

In this consecutive series of 30 children treated by microsurgical resection, complete resection was achieved in every patient and functional outcome improved significantly over follow-up, reaching a favorable mRS (0–2) in 76.7% with no survivor deteriorating (*p* < 0.0001). IVH (OR 28.5; *p* = 0.002), lower admission GCS (*p* = 0.0006), higher Supplemented S–M grade (*p* = 0.019), and postoperative complications (*p* = 0.016) were the variables significantly associated with poor outcome, while perioperative mortality was confined to eloquently located malformations (*p* = 0.037).

## 4. Discussion

The present study demonstrates that microsurgical resection of brain AVMs can achieve excellent anatomical and functional outcomes, with complete resection in all patients and a favorable functional outcome in more than three-quarters of the cohort at follow-up. Our findings further suggest that successful AVM surgery should not be evaluated solely by angiographic cure: while complete nidus resection was consistently achieved, long-term neurological outcome appeared to be associated primarily with the severity of the initial hemorrhagic insult—particularly IVH—together with neurological status at presentation and lesion complexity. Because the analysis is retrospective and univariable, these relationships represent associations rather than proven causal determinants and should be interpreted as exploratory and hypothesis-generating throughout the following discussion.

### 4.1. Surgical Efficacy and Functional Recovery

Complete resection remains the principal goal of AVM treatment because it immediately eliminates the risk of future hemorrhage. In the present cohort, microsurgical resection achieved a 100% resection rate, placing our results at the upper end of those reported in contemporary pediatric series. Similar findings were reported by Hsiao et al., who achieved complete resection in all surgically treated patients, while radiosurgery resulted in considerably lower resection rates and required prolonged latency before cure was achieved [6]. Likewise, Deng et al. reported excellent long-term outcomes after microsurgical treatment of 111 pediatric hemorrhagic AVMs, further supporting surgery as the most reliable curative modality in appropriately selected children [7].

Beyond anatomical cure, substantial neurological recovery was observed during follow-up. Our favorable outcome rate of 76.7% is comparable to the 82% neurological independence reported by Deng et al. and by Lim et al. [7,8]. Sanchez-Mejia et al. demonstrated that children experienced significantly better outcomes than adults after AVM resection despite comparable lesion characteristics, rupture rates, and treatment strategies, leading the authors to propose developmental neuroplasticity as the most plausible explanation [9]. The substantial functional improvement observed in our cohort is consistent with this concept; however, because no functional, structural, or advanced neuroimaging assessment of cortical reorganization was performed, developmental neuroplasticity remains a plausible hypothesized mechanism rather than a directly demonstrated finding in the present study.

### 4.2. Hemorrhagic Presentation

Hemorrhage represented the dominant mode of presentation in our series, occurring in approximately two-thirds of patients, consistent with the previous pediatric AVM literature [5]. The clinical importance of hemorrhage in children extends beyond the immediate neurological consequences: Fullerton et al. demonstrated that even relatively modest annual hemorrhage rates translate into a substantial lifetime probability of bleeding, supporting definitive treatment whenever feasible [3].

### 4.3. Intraventricular Hemorrhage and Functional Outcome

The most important finding of the present study was the identification of IVH as the variable most strongly associated with poor functional outcome, with an OR of 28.5—exceeding that of lesion complexity, hemorrhagic presentation, deep venous drainage, or eloquent location. This observation is supported by emerging pediatric literature: Guo et al. demonstrated that AVMs were the underlying cause in two-thirds of pediatric IVH cases and that greater ventricular blood burden was associated with significantly worse outcomes [15], while Neidert et al. incorporated intraventricular extension into the AVICH grading system because of its substantial influence on prognosis [16]. These findings suggest that IVH is not merely a marker of bleeding severity and may be an additional contributor to neurological injury through mechanisms including acute hydrocephalus, elevated intracranial pressure, impaired CSF circulation, and secondary ventricular inflammation [15,16,17].

Our findings therefore suggest that the anatomical distribution of hemorrhage may be more important than the mere presence of hemorrhage. Ventricular extension may represent a distinct pathophysiological entity characterized by acute hydrocephalus, CSF circulation impairment, and secondary inflammatory injury, all of which may contribute to long-term neurological disability. However, because IVH is closely interrelated with a lower admission GCS, acute hydrocephalus, and overall hemorrhage severity, and because no multivariable adjustment was possible in this small cohort, it cannot be determined whether IVH is an independent prognostic factor or simply a marker of a more severe initial brain injury. This association should therefore be regarded as hypothesis-generating and requires confirmation in larger multivariable analyses.

### 4.4. Admission Neurological Status and Lesion Complexity

Admission neurological status was another major factor associated with outcome. Lower GCS scores were strongly associated with worse functional recovery, consistent with findings by Deng et al., who identified pretreatment neurological status as an independent predictor of long-term disability after pediatric AVM surgery [7]. These findings suggest that outcome is influenced not only by successful resection but also by the extent of primary and secondary brain injury present before definitive treatment.

Higher Supplemented S–M grades were also associated with worse outcomes, consistent with the established role of AVM grading systems in estimating surgical risk and reflecting the combined effects of lesion size, venous drainage patterns, eloquence, and operative complexity. Meling and Patet emphasized that deep-seated and anatomically complex AVMs remain among the most challenging lesions in pediatric cerebrovascular surgery and frequently require individualized treatment strategies [10]. Postoperative complications were also associated with poor outcome; however, these events occur after treatment and may lie on the causal pathway between the initial injury and the final outcome rather than representing independent baseline prognostic factors, and should be interpreted accordingly.

### 4.5. Mortality and Eloquent Location

All deaths in our cohort occurred in patients harboring AVMs within eloquent brain regions, emphasizing the critical role of lesion location in determining surgical risk. The confinement of mortality to eloquently located AVMs is clinically noteworthy and is consistent with findings by Meling and Patet [10] and Shtaya et al. [5], who identified eloquent location as a key factor influencing outcome. Despite these risks, the overall outcomes remained favorable, consistent with findings by Hsiao et al. [6], suggesting that successful microsurgical management can still provide durable angiographic obliteration and meaningful neurological recovery when performed in experienced cerebrovascular centers. Because only four deaths were observed, the apparent confinement of mortality to eloquent-territory malformations is based on very small numbers and should be regarded as an exploratory observation rather than an established determinant of mortality.

### 4.6. Recurrence and Long-Term Surveillance

No recurrence or rebleeding was observed during the available follow-up period. Nevertheless, Oulasvirta et al. reported a recurrence rate approaching 5% despite angiographically confirmed cure, while Lang et al. demonstrated that surveillance imaging can identify clinically silent recurrences years after surgery [11,12]. These observations challenge the traditional assumption that angiographic cure is synonymous with permanent cure in pediatric patients. Long-term radiological surveillance should therefore remain an integral component of pediatric AVM management.

### 4.7. Limitations

Several limitations should be acknowledged. First, this was a retrospective single-center study with a relatively small sample size (*n* = 30), limiting statistical power and generalizability. To our knowledge, this represents the first dedicated microsurgical series of pediatric brain AVMs from the Western Balkans. While the sample size is limited by the rarity of the condition in a single-center setting, the absence of any previous published comparable regional series underscores both the novelty of these data and the need for prospective multicenter collaboration in this geographic area. Expanding the cohort within a single institution was not feasible given the rarity of pediatric AVMs; a prospective multicenter registry is the appropriate strategy to increase statistical power and to enable the multivariable modeling that the present sample size precludes. Second, the low number of poor-outcome events precluded reliable multivariable modeling, and all identified predictors should be interpreted as univariable associations. Third, postoperative resection was confirmed primarily by CTA rather than systematic DSA, which may have reduced sensitivity for detecting small residual nidus components. Finally, follow-up duration was insufficient to fully assess late AVM recurrence, an important consideration in pediatric populations. No a priori power calculation was performed, and with only seven poor-outcome events and four deaths, the study is underpowered, so the wide confidence intervals (for example, OR 28.5; 95% CI 2.65–306.6 for IVH) indicate that the reported odds ratios are exploratory rather than precise effect estimates. All analyses are univariable, so residual and unmeasured confounding cannot be excluded and no variable can be established as an independent predictor. In particular, adjustment for Spetzler–Martin grade, age, and postoperative complications was not possible, so it cannot be determined whether any single factor remains independently associated with outcome after mutual adjustment. Because the cohort was restricted to children undergoing primary microsurgical resection, patients treated with radiosurgery, embolization, or multimodality strategies were not represented, which limits external validity to surgically selected patients managed at high-volume cerebrovascular centers. Multiple univariable comparisons were performed without formal correction, increasing the risk of false-positive associations. In addition, because the Supplemented Spetzler–Martin grade incorporates patient age and hemorrhagic (versus unruptured) presentation, it partially overlaps with the separately analyzed variables of hemorrhagic presentation and intraventricular hemorrhage, so the associations of these related variables with outcome cannot be regarded as mutually independent. Moreover, postoperative complications, although analyzed alongside baseline variables, may represent intermediate events on the causal pathway between the initial insult and the final outcome rather than independent baseline prognostic factors, and their association with poor outcome should be interpreted in that light. Functional outcome was scored retrospectively and without blinding, and interobserver agreement was not assessed. Complete resection was confirmed primarily by CTA rather than by systematic DSA, and the 6–9-month follow-up is too short to exclude the late recurrence that is well documented in children. Accordingly, the present findings should be regarded as exploratory and hypothesis-generating.

## 5. Conclusions

Microsurgical resection of pediatric brain AVMs achieved complete resection in all patients and was associated with substantial functional recovery, with favorable outcomes in more than three-quarters of the cohort. While complete angiographic resection was consistently achieved, long-term functional outcome appeared to be associated primarily with the severity of the initial hemorrhagic insult. In this small retrospective surgical cohort, IVH showed the strongest association with poor functional outcome, and lower admission GCS and higher Supplemented S–M grade were likewise associated with worse recovery; because the analysis was univariable, these variables should be regarded as associated with—rather than as independent determinants of—outcome.

Despite a high prevalence of hemorrhagic presentation, most survivors demonstrated significant neurological improvement during follow-up, underscoring the remarkable recovery potential of the pediatric brain. The confinement of all deaths to eloquently located AVMs highlights the continuing importance of lesion anatomy in surgical risk assessment.

In pediatric AVMs, angiographic cure does not by itself guarantee neurological recovery; rather, IVH, neurological status at presentation, and lesion complexity appear to be associated with the eventual extent of recovery. Because these observations derive from a small, single-center, surgically selected cohort with limited follow-up, they should not be generalized beyond similar high-volume neurosurgical centers, and larger multicenter prospective studies incorporating multivariable analysis and longer angiographic surveillance are required before robust prognostic conclusions can be drawn.

## Figures and Tables

**Table 1:** Baseline demographic and clinical characteristics of the pediatric AVM cohort (*n* = 30).

Characteristic	Value	%/Dispersion
Age, years—mean ± SD	13.6 ± 3.7	Range 4–18
Age, years—median (IQR)	14	11.3–17.0
Sex—male	18	60.0%
Sex—female	12	40.0%
GCS on admission—median (IQR)	15	13.75–15.0
Severe GCS (3–8) *	6/28	21.4%
Any hemorrhagic presentation	19	63.3%
Intracerebral hemorrhage (ICH)	18	60.0%
Intraventricular hemorrhage (IVH)	10	33.3%
Combined IC + IV hemorrhage	9	30.0%
Epilepsy (any type)	12	40.0%
Generalized tonic–clonic seizure	8	26.7%
Partial seizure	4	13.3%
Headache	17	56.7%
Focal neurological deficit	12	40.0%

SD, standard deviation; IQR, interquartile range; GCS, Glasgow Coma Scale; IC, intracerebral; IV, intraventricular. * Denominator = 28 patients with a documented admission GCS (2 missing).

**Table 2:** Morphological and angioarchitectural characteristics of the AVM nidus.

Characteristic	*n*	% (Denominator)
Side—right	16	53.3%
Side—left	14	46.7%
Nidus size < 3 cm	14	46.7%
Nidus size 3–6 cm	13	43.3%
Nidus size > 6 cm	2	6.7%
Supp. S–M grade—median (range)	3.5	2–8
Supp. S–M grade 2	1	3.3%
Supp. S–M grade 3	13	43.3%
Supp. S–M grade 4	6	20.0%
Supp. S–M grade 5	5	16.7%
Supp. S–M grade 6	2	6.7%
Supp. S–M grade 8	1	3.3%
Supp. S–M grade not documented	2	6.7%
Eloquent location (any)	14	46.7%
Frontal nidus	8	26.7%
Parietal nidus	7	23.3%
Occipital nidus	6	20.0%
Deep nidus	6	20.0%
Motor-cortex nidus	4	13.3%
MCA arterial feeder	16	66.7%
PCA arterial feeder	10	41.7%
Pericallosal feeder	8	33.3%
ACA feeder	7	29.2%
Deep venous drainage (deep or combined)	13	43.3%
Venous ectasia	8/21	38.1%

MCA, middle cerebral artery; PCA, posterior cerebral artery; ACA, anterior cerebral artery; Supp. S–M, Supplemented Spetzler–Martin. Lobar/feeder categories are non-mutually exclusive. Feeder counts were documented in 24 patients; venous-phase features were seen in 21–27 patients as indicated.

**Table 3:** Surgical procedures performed and postoperative complications *(n* = 30).

Procedure/Complication	*n*	%
Microsurgical AVM resection	30	100.0%
Intracerebral hematoma evacuation	13	43.3%
External ventricular drainage	4	13.3%
Ventriculoperitoneal shunt	3	10.0%
Any postoperative complication	6	20.0%
Postoperative intracerebral hemorrhage	5	16.7%
Meningitis	2	6.7%
Surgical-site (wound) infection	2	6.7%
Shunt malfunction	1	3.3%

Procedure categories are non-mutually exclusive (a single patient may have undergone more than one procedure).

**Table 4:** Distribution of the modified Rankin Scale (mRS) at discharge and at 6–9 months.

mRS	Discharge *n*	Discharge %	6–9 mo *n*	6–9 mo %	Category
0	3	10.0	11	36.7	Favorable
1	8	26.7	8	26.7	Favorable
2	4	13.3	4	13.3	Favorable
3	2	6.7	3	10.0	Poor
4	8	26.7	0	0.0	Poor
5	1	3.3	0	0.0	Poor
6 (death)	4	13.3	4	13.3	Death
Favorable (0–2)	15	50.0	23	76.7	—
Poor (3–6)	15	50.0	7	23.3	—

mRS, modified Rankin Scale. The four perioperative deaths are carried forward as mRS 6 at follow-up. Wilcoxon signed-rank test, discharge vs. 6–9 months: *p* < 0.0001.

**Table 5:** Univariable variables associated with poor functional outcome (mRS 3–6) at 6–9 months. Statistically significant results (*p* < 0.05) are shown in bold.

Variable	Poor (*n* = 7)	Good (*n* = 23)	OR (*p*-Value)
Intraventricular hemorrhage	6/7 (85.7%)	4/23 (17.4%)	28.5 (*p* = 0.002)
Lower admission GCS †	Median 8	Median 15	— (*p* = 0.0006)
Any complication	4/7 (57.1%)	2/23 (8.7%)	14.0 (*p* = 0.016)
Postoperative re-hemorrhage	3/7 (42.9%)	2/23 (8.7%)	7.9 (*p* = 0.068)
Supp. S–M grade (continuous) †	Median 5	Median 3	— (*p* = 0.019)
Supp. S–M grade ≥ 5	4/7 (57.1%)	4/23 (17.4%)	6.33 (*p* = 0.060)
Deep venous drainage	5/7 (71.4%)	8/23 (34.8%)	4.69 (*p* = 0.190)
Hemorrhagic presentation	6/7 (85.7%)	13/23 (56.5%)	4.62 (*p* = 0.215)
Eloquent location	5/7 (71.4%)	9/23 (39.1%)	3.89 (*p* = 0.204)
Nidus size ≥ 3 cm	4/7 (57.1%)	11/23 (47.8%)	1.45 (*p* = 1.00)

OR, odds ratio (Fisher’s exact test for categorical predictors); GCS, Glasgow Coma Scale; Supp. S–M, Supplemented Spetzler–Martin. † For continuous predictors the comparison is by Mann–Whitney U test; group medians are shown.

**Table 6:** Variables associated with perioperative mortality (4 deaths among 30 patients).

Variable	Effect Estimate	*p*-Value
Eloquent location	4/14 vs. 0/16	0.0365
Lower admission GCS	Median 9 vs. 15	0.0324
Intraventricular hemorrhage	OR 8.14	0.0952
Supplemented S–M grade	Mean 5.25 vs. 3.71	0.1010
Deep venous drainage	OR 4.80	0.2903
Hemorrhagic presentation	OR 1.88	1.0000

Categorical predictors: Fisher’s exact test (OR reported). Continuous predictors: Mann–Whitney U test (group medians reported). GCS, Glasgow Coma Scale; OR, odds ratio; S–M, Spetzler–Martin.

## Data Availability

The data that support the findings of this study are available from the corresponding author upon reasonable request. The data are not publicly available due to privacy and ethical restrictions.
